# Reducing delays to multidrug-resistant tuberculosis case detection through a revised routine surveillance system

**DOI:** 10.1186/s12879-020-05298-8

**Published:** 2020-08-12

**Authors:** Basra Esmail Doulla, Stephen Bertel Squire, Eleanor MacPherson, Esther Stanslaus Ngadaya, Beatrice Kemilembe Mutayoba, Ivor Langley

**Affiliations:** 1 Ministry of Health, Community Development, Gender, Elderly and Children; National Tuberculosis and Leprosy Programme, Dar es Salaam, Tanzania; 2 Liverpool School of Tropical Medicine, Centre for Applied Health Research and Delivery, Liverpool, UK; 3 National Institutes for Medical Research, Muhimbili Medical Research Centre, Dar es Salaam, Tanzania

**Keywords:** Reference laboratory, Routine surveillance system, Previously treated cases, Specimens, Tuberculosis

## Abstract

**Background:**

Implementation of an effective Tuberculosis Routine Surveillance System in low-income countries like Tanzania is problematic, despite being an essential tool for the detection and effective monitoring of drug resistant tuberculosis. Long delays in specimen transportation from the facilities to reference laboratory and results dissemination back to the health facilities, result in poor patient management, particularly where multidrug-resistant tuberculosis disease is present.

**Methods:**

Following a detailed qualitative study, a pilot intervention of a revised Tuberculosis Routine Surveillance System was implemented in Mwanza region, Tanzania. This included the use of rapid molecular methods for the detection of both tuberculosis and drug resistance using Xpert MTB/RIF in some Mwanza sites, the use of Xpert MTB/RIF and Line Probe Assay at the Central Tuberculosis Reference Laboratory, a revised communication strategy and interventions to address the issue of poor form completion. A before and after comparison of the intervention on the number of drug resistant tuberculosis cases identified and the time taken for results feedback to the requesting site was reported.

**Results:**

The revised system for previously treated cases tested at the Central Reference Laboratory was able to obtain the following findings; the number of cases tested increased from 75 in 2016 to 185 in 2017. The times for specimen transportation from health facilities to the reference laboratory were reduced by 22% (from 9 to 7 days). The median time for the district to receive results was reduced by 36% (from 11 to 7 days). Overall the number of drug resistant tuberculosis cases starting treatment increased by 67% (from 12 to 20).

**Conclusion:**

Detection of drug resistance could significantly be enhanced, and delays reduced by introduction of new technologies and improved routine surveillance system, including better communication using mobile applications such as ‘WhatsApp’ and close follow-ups. A larger scale study is now merited to ascertain if these benefits are robust across different contexts.

## Background

Tuberculosis (TB) is the highest single cause of death worldwide from an infectious disease and continues to be a major public health problem [1]. The World Health Organization (WHO) estimates, 10.4 million people contracted TB in 2016 of which 4 million of these cases were undiagnosed. 480,000 were estimated to be cases with TB that is resistant to at least Isoniazid (INH) and Rifampicin (RIF), with or without resistance to other first-line drugs (MDR-TB) cases of which 6.2% were Extensively Drug-Resistant TB (XDR-TB). 1.3 million people were estimated to have died of the disease [1]. In a resource-constrained setting, diagnosis of MDR-TB can take many weeks caused by long delays in starting and completing diagnosis with limited diagnostic facilities that can detect drug-resistance [2]. Delay in diagnosis and treatment has serious consequences for disease control at both the individual and the community levels [3]. The strength of the TB laboratory network is often a direct reflection of the success of the TB Control Programmes [4]. Poor TB case detection and rising TB drug resistant are in part the result of historically neglected laboratory services, slow technology transfer, and a lack of new more accurate TB diagnostic tools [5]. The WHO recommends rapid and sensitive diagnostic methods that provide information on drug resistance (i.e. methods such as GeneXpert (Xpert MTB/RIF) and Line-Probe Assays (LPA) [6]). The LPA Genotypic (molecular) tests for identification of isoniazid and rifampicin resistance to first-line anti-TB drugs [7], and Genotype® MTBDRplus assay have shown promise for the diagnosis of drug resistant TB for the second line (SL-LPA) anti TB drugs. These tests can be performed in a single working day and detect the presence of mutations associated with drug resistant TB [8, 9]. Xpert can be used as a point of care technology in some contexts and can reduce the time between specimen collection and diagnostic result [10].

**Fig. 1:**
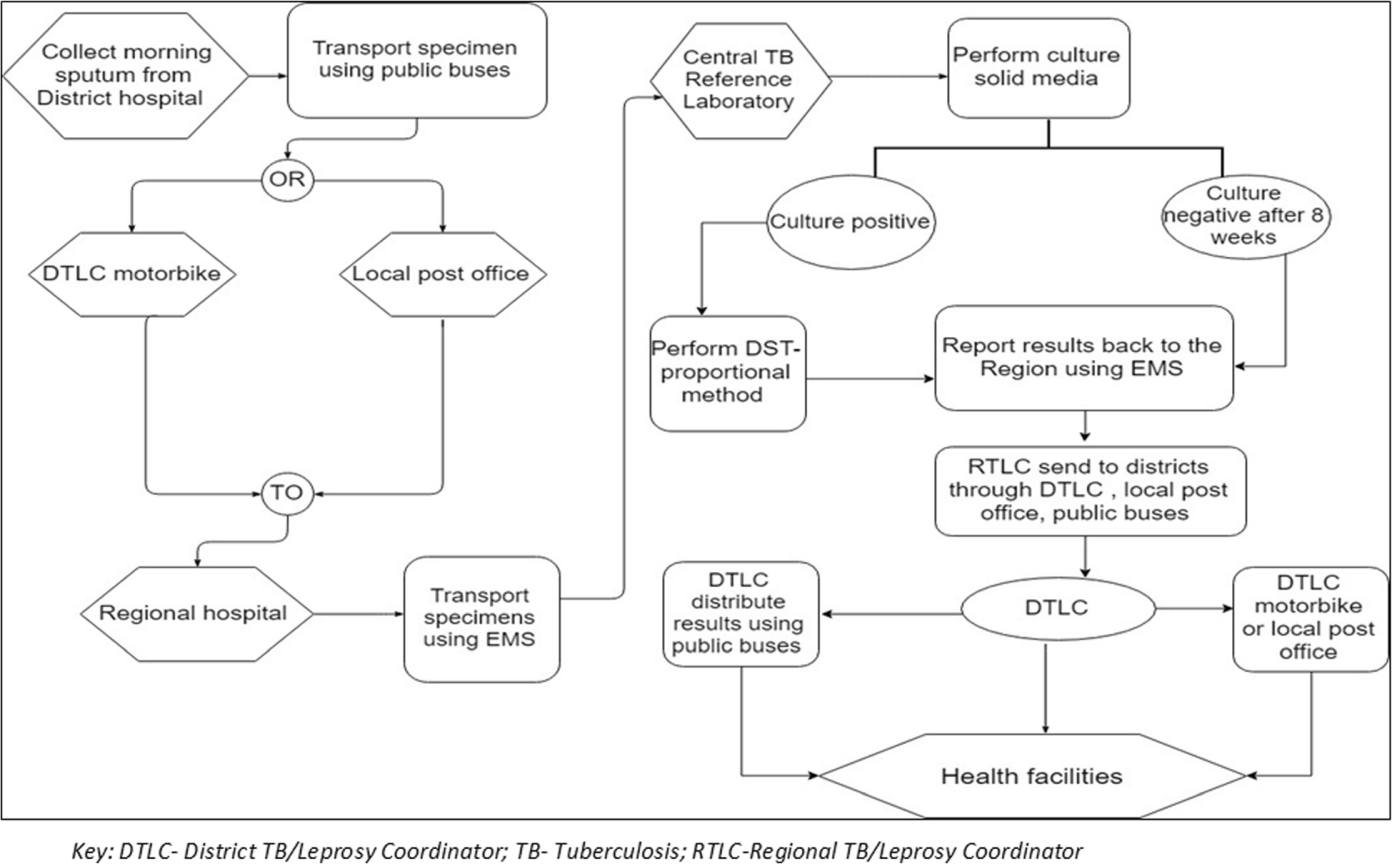
Specimen flow for the existing Routine Surveillance System

A successful continuous Routine Surveillance System (RSS) is an important element in the process of detecting and monitoring drug resistant TB. The existing RSS of the National TB and Leprosy Program (NTLP) in Tanzania shown in Fig. 1, specifies sputum specimens should be submitted to the Central TB Reference Laboratory (CTRL) for culture and drug susceptibility testing (DST) for 25% of all new TB cases and 100% of previously treated TB cases [11]. However, these percentages for DST at the CTRL have historical not been achieved. Indeed for previously treated TB only 61% of cases were sent for testing even in the best year of the three-year period investigated, suggesting cases of drug resistance are likely to have been missed [12]. This study focused on the previously treated TB cases because that is where most MDR TB cases are found [13, 14]. Failure to appropriately track these cases could lead to the emergence of XDR-TB. Qualitative findings showed that the system of TB specimen transportation in Tanzania was a major problem in remote health facilities as there were no reliable and frequent means of transport [12]. The existing RSS was underperforming in relation to the feedback and communication of the drug susceptibility testing results from the CTRL to the peripheral health facilities [12]. Based on these findings, in this study a revised routine surveillance system was designed and piloted in Mwanza region, Tanzania. The design took account of the weaknesses identified from the earlier qualitative study [12] and changes in the availability of molecular diagnostic techniques. A ‘before and after’ comparison was conducted of the performance of the RSS. Measurements were taken to assess the effect of the revised RSS on the number of specimens received and tested for drug susceptibility, the time for specimen transportation and results feedback, and the number of MDR-TB cases detected.

Consequently, a study designed to understand the potential effects of a revised routine surveillance system for previously treated TB cases on delays and level of MDR-TB diagnosis by piloting the revised approach in Mwanza region, Tanzania.

## Methods

### Study site

The study was carried out in four districts of Mwanza region (population of 2.77 million with high burden of HIV/AIDS [14]) in Tanzania from 1st March 2017 to 1st March 2018. Mwanza was chosen because it is a remote area with a high TB case load and therefore an area where transportation of specimens is particularly challenging. At the time of conducting this study, there were eighty smear microscopy diagnostic centres, with five centres installed with Xpert for routine diagnosis. The region is 1142 km by road from Dar es Salaam where the CTRL is located where Drug Susceptibility testing currently takes place. Due to its size and location piloting the revised RSS in Mwanza was considered an effective means of evaluating impact of the revised RSS.

### Study design and population

A prospective pilot study of the revised RSS for previously treated TB cases was conducted with ‘before and after’ quantitative analyses of impact.

### Study procedure

#### Revised routine surveillance system

**Fig. 2:**
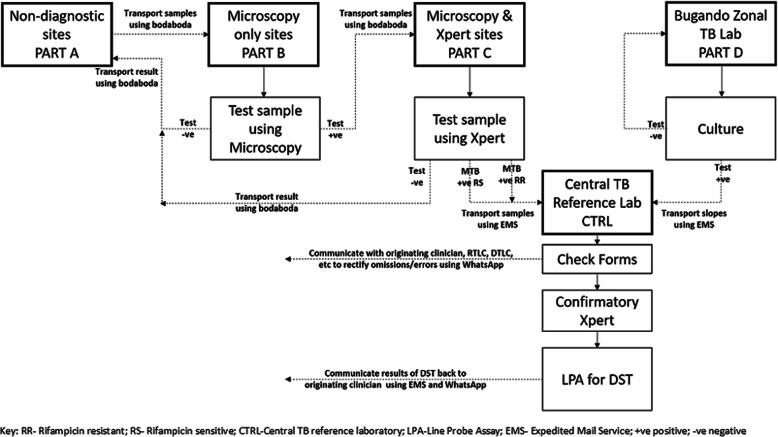
Specimen flow for the revised Routine Surveillance System

The current (‘Before’) RSS is described in Fig. 1. In the revised RSS (‘After’), sputum specimens were collected from four study sites (shown in Fig. 2) and were examined using the molecular method in a modified algorithm for detection of drug resistant TB. The algorithm design was in two parts. Part “A” for sites without Xpert capacity where smear microscopy was used as the primary tool for TB diagnosis, or a site where neither microscopy nor Xpert MTB/RIF were available known as non-diagnostic centres. If tests undertaken in the peripheral laboratory were smear positive or could not be tested, then a sputum specimen was transported to the nearest Xpert site. Part “B” for sites where an Xpert had been installed and was used as the primary diagnostic tool, where only specimens that were detected as Rifampicin resistant were sent to the CTRL for confirmatory LPA testing (Fig. 2).

#### Specimen collection, transportation and processing

At Peripheral sites in Mwanza, specimens collected from previously treated cases in non-diagnostic sites were transported to microscopy sites for diagnosis. Specimens collected at sites with microscopy together with those received from non-diagnostic sites were examined by Light Emitting Diode (LED) microscopy. Microscopy results were communicated to the clinician for patient management. Positive smear specimens were then transported to the nearest Xpert site using motorbikes commonly known as *“bodaboda”*. Specimens were not batched before transportation as with the existing RSS, as it had been noted this could lead to many days delays. The bodaboda was chosen as it was seen as a reliable transportation system in the peripheries and was identified and registered in collaboration with Koninklijke Nederlandse Centrale Vereniging tot bestrijding der Tuberculose (KNCV).

At the five Xpert sites in Mwanza, specimens were analysed using Xpert. When rifampicin resistance was detected the specimen was sent to the CTRL by the Expedited Mail Services (EMS), without batching, to be tested for both first and second-line anti TB drugs using LPA. This was communicated to the CTRL through WhatsApp groups for easy tracking and sharing information in case of any problems (Fig. 2). The WhatsApp groups were initiated for easy communication due to the lack of official telephone lines and internet connectivity at the Mwanza study sites and the CTRL. The WhatsApp was instantaneous, affordable and reachable to all at any time. Various groups were created by the NTLP Manager such as *Vitendanishi* for supplies; to follow up laboratory supplies availability, stock status and any other issues regarding the TB laboratory services across the country. The *MSD group for* Medical Store Department; for tracking any orders requested through NTLP. The WhatsApp groups facilitated the resolution of any concerns in a timely manner and eased results dissemination, consequently shortening both transit and overall turnaround time.

On arrival at the CTRL, the specimens with their accompanying revised forms were checked for completeness and accuracy against the specimen container. Missing information was communicated to the requester immediately to rectify the identified problems. All specimen examination procedures were carried out in a Biosafety cabinet class II. Results were recorded in the TB laboratory registers and on the TB laboratory request forms. The forms were immediately sent back to the requesters through EMS.

#### Summary of interventions included in the revised RSS

In summary, the key difference in revised RSS are: changes in diagnostic approach by using Xpert at the local site in Mwanza and Xpert and LPA at the CTRL; introduction of reliable specimen’s transportation from the remote sites in Mwanza using Bodaboda; changes in results dissemination to the requester including copies to the Regional TB and Leprosy Coordinator (RTLC); close monitoring of specimen transportation time by the CTRL with expectation of a minimum of 4 days and maximum of 7 days; comparison of the number of specimens received at the CTRL versus cases notified and communicated to the RTLC; TB laboratory request forms were revised to make them clearer and the TB laboratory register modified to accommodate the changes; supportive supervision and mentoring schedule was created and shared with the Mwanza team; improved communication between the CTRL and the health facilities in Mwanza region through WhatsApp groups “Mwanza family”, RTLC, DTLCs and CTRL staff; all specimen transportation used a system where a specimen is inserted in a clear zip bag and put in a small plastic container with a screw cap (primary container) together with absorbent material in case of leakages. This is placed in a plastic container (secondary container) together with the completed TB laboratory request forms and then put in either a metal, plastic or cardboard box [15, 16] know as a Triple pack and specimens were transported to the intended study site on the day of collection whenever possible even if it was just a single specimen. To support all this training on the revised RSS was conducted at all study sites in Mwanza and the CTRL.

The primary outcomes of the study were the impact of the revised RSS on specimen transit time and turnaround time. Routine NTLP data from 2015 were used to determine the sample size. The study sample size was powered based on the turnaround outcome. A total of 315 specimens were required to show a significant reduction in this time using a 95% confidence interval, 80% power, and hypothesised average difference of 10 days with a population standard deviation of 44.88.^1^

Population standard deviation of 44.8^1^

**Table:** 

Estimated Turnaround time for Mwanza Region for the year 2015					
TAT calculation = time from specimen receipt at CTRL to the time results were reported and communicated back to the requester					
Year	N (TAT)	mean (TAT)	sd (TAT)	min (TAT)	max (TAT)
2015	69	88.87	44.88	12	304

***Key:*** *N- Number; TAT- turnaround time; CTRL- Central Tuberculosis Reference Laboratory; sd- standard deviation.*

### Formula

$$ \mathrm{n}=\left(\mathrm{Z}\alpha /2+\mathrm{Z}\beta \right)\;2\ast 2\ast \sigma 2/\mathrm{d}2 $$

Where;

Zα/2 is the critical value of the Normal distribution at α/2 = 1.96.

Zβ is the critical value of the Normal distribution at β = 0.84.

σ2 is the population variance.

d is the difference you would like to detect.

Sample size titration.

**Table:** 

Z5%/2 = 1.96	Z20% = 0.84	sd	d	n
1.96	0.84	44.8	5	1259
1.96	0.84	44.8	10	315
1.96	0.84	44.8	15	140
1.96	0.84	44.8	20	79

### Data collection and analysis

Data were double entered independently into computer database using Epidata software version 3.1, by two data entrants, internal data consistency checks were built into the database. Validation and consistency checks were done by the statistician using SPSS version 17. Queries were provided to the Data entrants for verification and rectification and errors were corrected before analysis.

Analysis was performed on data collected from March 2017 to March 2018 in Mwanza and the CTRL. Transit time defined as time from specimen collection to the time the specimen is received at the CTRL and turnaround time defined as the time from specimen receipt to the time results were reported and communicated back to the requester were reported.

### Ethical statement

The study was approved by the Medical Research Coordinating Committee of the National Institute for Medical Research in Tanzania, (NIMR); reference no. NIMR/HQ/R.8a/Vol. IX/2347 and the Liverpool School of Tropical Medicine (LSTM). No oral or written consents were obtained.

## Results

### Results before pilot interventions implemented 2016/2017

There were 75 Mwanza specimens analysed at the CTRL from March 2016 to March 2017, of these 48 (64%) were analysed, 27 (36%) had missing information and could not be analysed. No positive isolate specimens were received from the Zonal culture laboratory in Mwanza (Table 2). The transit time recorded for both sputum and isolates showed 44% of specimens were received after 21 days. The Interquartile Ranges cut off points were 25th First Quartile and 75th third Quartile. Overall the median and (interquartile range - IQR) was 12 (51) days in 2016/2017 (Table 2). The turnaround time had an overall median (IQR) of 62 (10) days (Table 4).

**Fig. 3:**
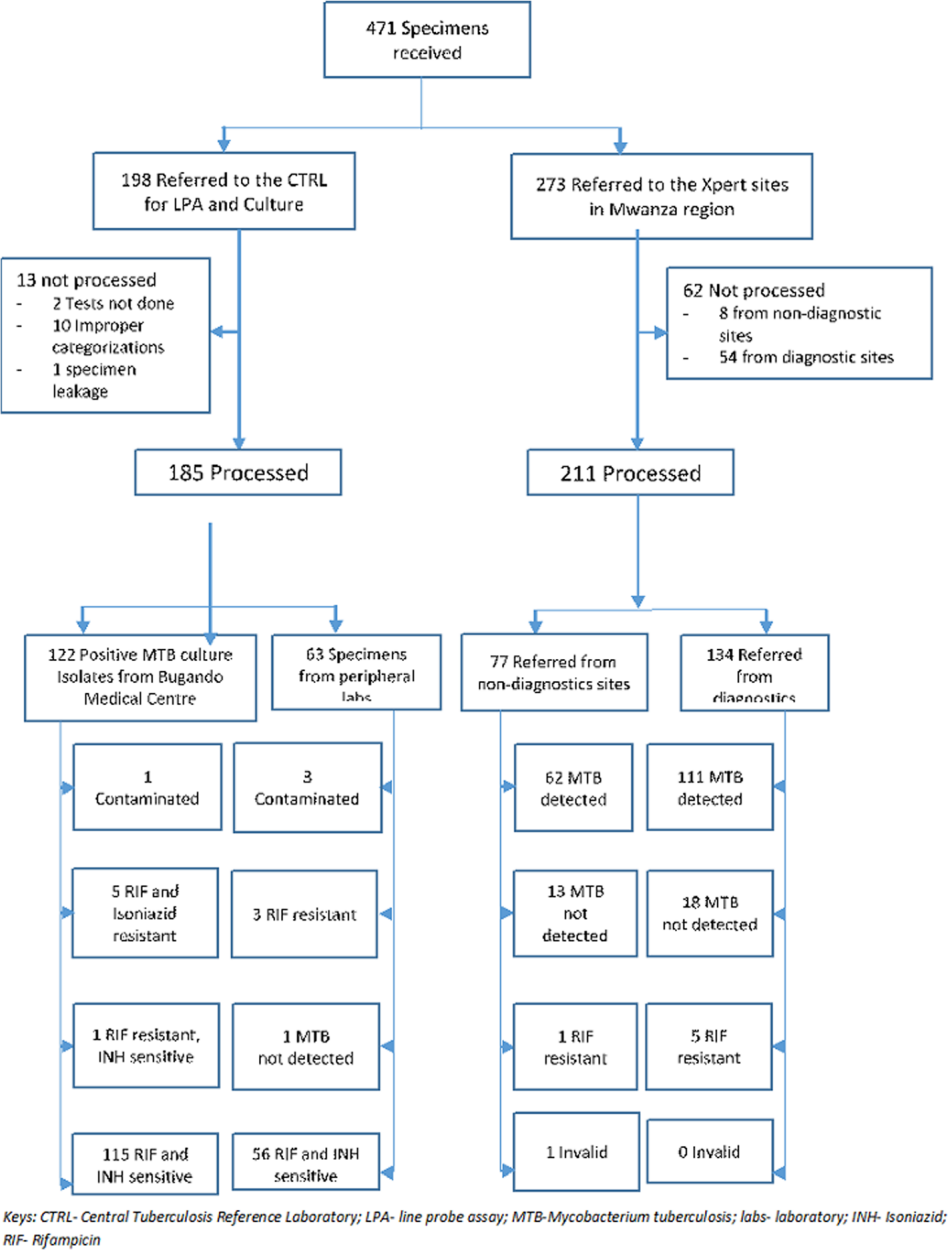
Specimens Analysed 2017/2018 – a pilot study

A total of 471 specimens were collected during the study period from March 2017 to March 2018 (2017/2018). 273 (58.0%) specimens were received in the Mwanza sites and 198 (42.0%) at the CTRL. Of those received at the CTRL, 185 (93.4%) were sent for examination with LPA. Of these 1 had no Mycobacterium TB detected and 5% of the uncontaminated specimens were found to be resistant to Rifampicin or Isoniazid or both (Fig. 3, Table 1).

**Table 1:** Drug susceptibility testing – Line Probe Assay at the CTRL

S/N	LPA – CTRL	Positive isolate - Zonal TB culture laboratory	Sputum specimens – peripheral laboratory	Total
1	RIF (R), INH (R)	5 (3%)	0 (0%)	5 (3%)
2	RIF (R)	1 (1%)	3 (2%)	4 (2%)
3	INH (R)	0 (0%)	0 (0%)	0 (0%)
4	Sensitive to both RIF and INH	115 (64%)	56 (31%)	171 (95%)
5	Total	121 (68%)	59 (33%)	180 (100%)
6	Contaminated	1 (1%)	3 (1%)	4 (2%)

Key: *LPA* Line Probe Assay, *TB* Tuberculosis, *CTRL* Central Tuberculosis Reference Laboratory, *RIF (R)* Rifampicin resistant, *INH (R)* Isoniazid resistant

**Fig. 4:**
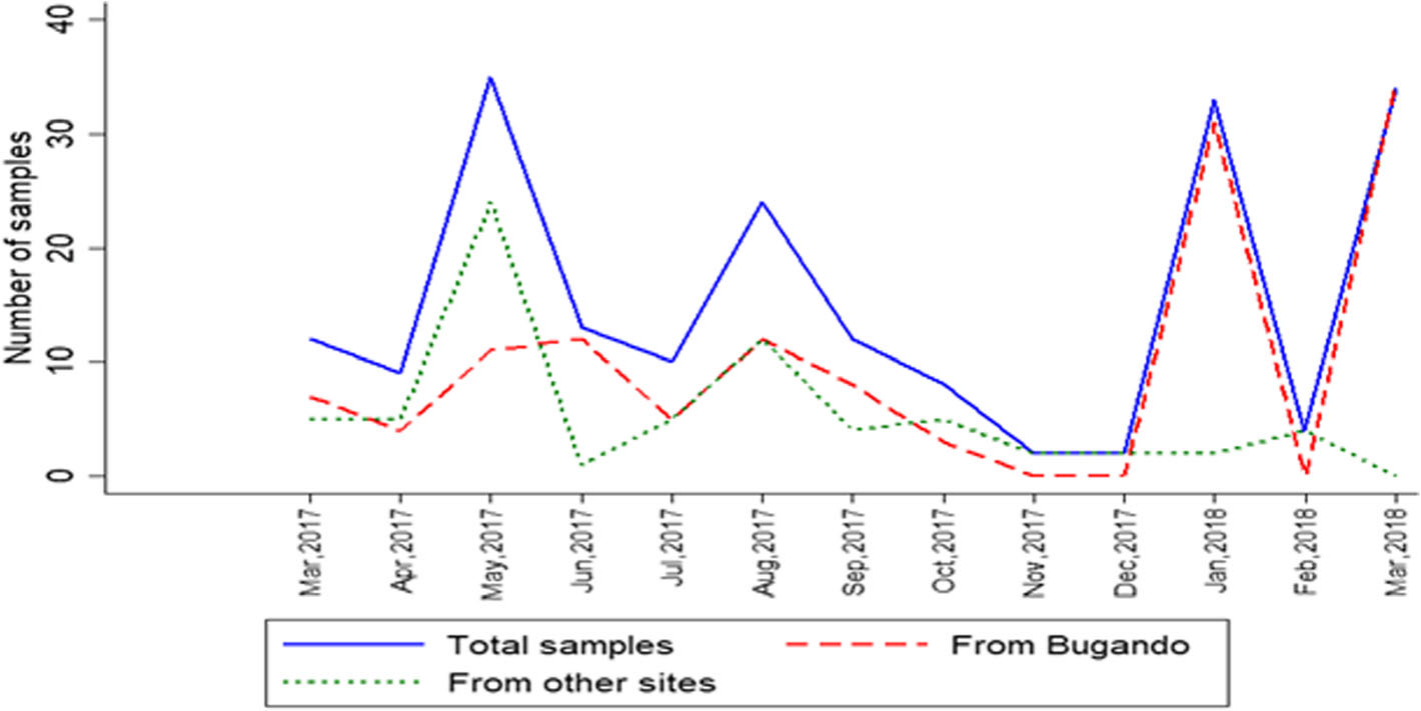
Summary number of previously treated patients’ specimens received at the CTRL

Figure 4 shows the variation in the number of specimens received at the CTRL throughout the study. This shows the largest number of specimens were recorded in May 2017, January 2018 and March 2018. Few specimens were received in April and December 2018 (Fig. 4).

In March 2017/2018 the total number of specimens that were examined at the CTRL was 185, of those 63 (34%) were sputum specimens from the periphery and 122 (66%) were Mycobacterium TB positive isolates from Zonal TB culture laboratory.

### Transit time (time from specimen collection to the time the specimen is received at the CTRL)

Transit time for both sputum and isolates ranged from 2 to 21 days and 21% of specimens were received after 21 days. The median and (interquartile range - IQR) transit time was 10 (9) days (Table 2). This compares to the median and (interquartile range - IQR) of 12 (51) days in 2016/2017.

**Table 2:** Transit time: Number (%) of the specimen received at CTRL from Mwanza site

Year	Specimen	< 3 days	> = 3 days & < 7 days	> = 7 days & < 21 days	> = 21 days	Total	1st IQR	3rd IQR	Median	IQR
2017/2018										
After Interventions	Sputum	3 (5%)	18 (29%)	39 (62%)	3 (5%)	63	6	9	7	3
	Isolate	0 (0%)	30 (25%)	56 (46%)	36 (30%)	122	7	34	10	27
	**Total**	**3 (2%)**	**48 (26%)**	**95 (52%)**	**39 (21%)**	**185**	**6**	**15**	**10**	**9**
2016/2017										
Before Intervention	Sputum	13 (27%)	4 (8%)	10 (21%)	21 (44%)	48	3	54	12	51
	Isolate	0 (0%)	0 (0%)	0 (0%)	0 (0%)	0				

Key: *IQR* Interquartile range = Third Quartile – First Quartile.

Out of 273 specimens referred to the Xpert sites in Mwanza region; 62 (23%) were rejected. 128 of the 151 (85%) sputum specimens were received in less than 3 days. In 2016/2017 no data was recorded for specimens referred to Xpert sites (Table 3).

**Table 3:** Transit time from Mwanza study Non Xpert to Xpert sites in 2017/2018

Year	Specimen	< 3 days (%)	> = 3 & < 7 days (%)	> = 7 & 21 days (%)	> = 21 days (%)	Rejected specimens (%)	Total
2017–2018	Sputum	128 (85)	5 (3)	1 (1)	1 (1)	16 (10)	151
	Isolate	75 (62)	1 (1)	0	0	46 (37)	122
Total		203 (74)	6 (2)	1 (0.5)	1 (0.5)	62 (23)	273

Key: Rejected specimens- leakage or empty container received

### Turnaround time

Turnaround time was calculated by subtracting the date results were reported from the date specimen was received at the CTRL. The overall median (IQR) turnaround time was 7 (8) days for 2017/2018 and for the 2016–2017, the median IQR was 62 (10) days. There was a significant (*p*-value < 0.001) decrease in turnaround time of 55 days after the intervention (Table 4).

**Table 4:** Turnaround times: Number (%) of specimens’ results dissemination from CTRL to Mwanza sites

Specimen	< 3 days	> = 3 & < 7 day	> = 7 & < 21 day	> = 21 days	Total	Median	IQR
2017/2018							
Sputum	11 (18%)	38 (63%)	8 (13%)	3 (5%)	60	3	2
Isolate	14 (12%)	21 (17%)	54 (45%)	31 (25%)	122	7	22
Total	25 (14%)	59 (33%)	62 (34%)	34 (19%)	182	7	8
2016/2017							
Sputum	0	0	0	8 (11)	8	62	10
Isolate	0	0	0	0	0	0	0
Total	0	0	0	8 (11)	0	62	10

Note: 2017/2018–3 specimens were not processed and 2016/2017–67 specimens had missing date hence excluded from the analysis

Key: *CTRL* Central tuberculosis reference laboratory, *IQR* Interquartile range.

### Turnaround time peripheral sites in Mwanza region

Out of 211 specimens processed, 59(28%) had missing turnaround times. Of the 152 to known turnaround times, 145 (95%) results were sent back in less than 3 days while 4 (3%) were sent back in more than 21 days (Table 5).

**Table 5:** Turnaround times: Number (%) of specimen results dissemination from Xpert site to Non-Xpert sites in Mwanza Region 2017/2018

Centre	< 3 days	> = 3 & < 7 day	> = 7 & < 21 day	> = 21 days	Total
Diagnostic	75 (96)	2 (3)	0 (0)	1 (1)	78
Non-Diagnostic	70 (95)	1 (1)	0 (0)	3 (4)	74
Total	145 (95)	3 (2)	0 (0)	4 (3)	152

*Note: 62 Specimens were excluded from the analysis*

Key: Diagnostic- sites with microscopy; Non-diagnostic- site without microscopy.

**Fig. 5:**
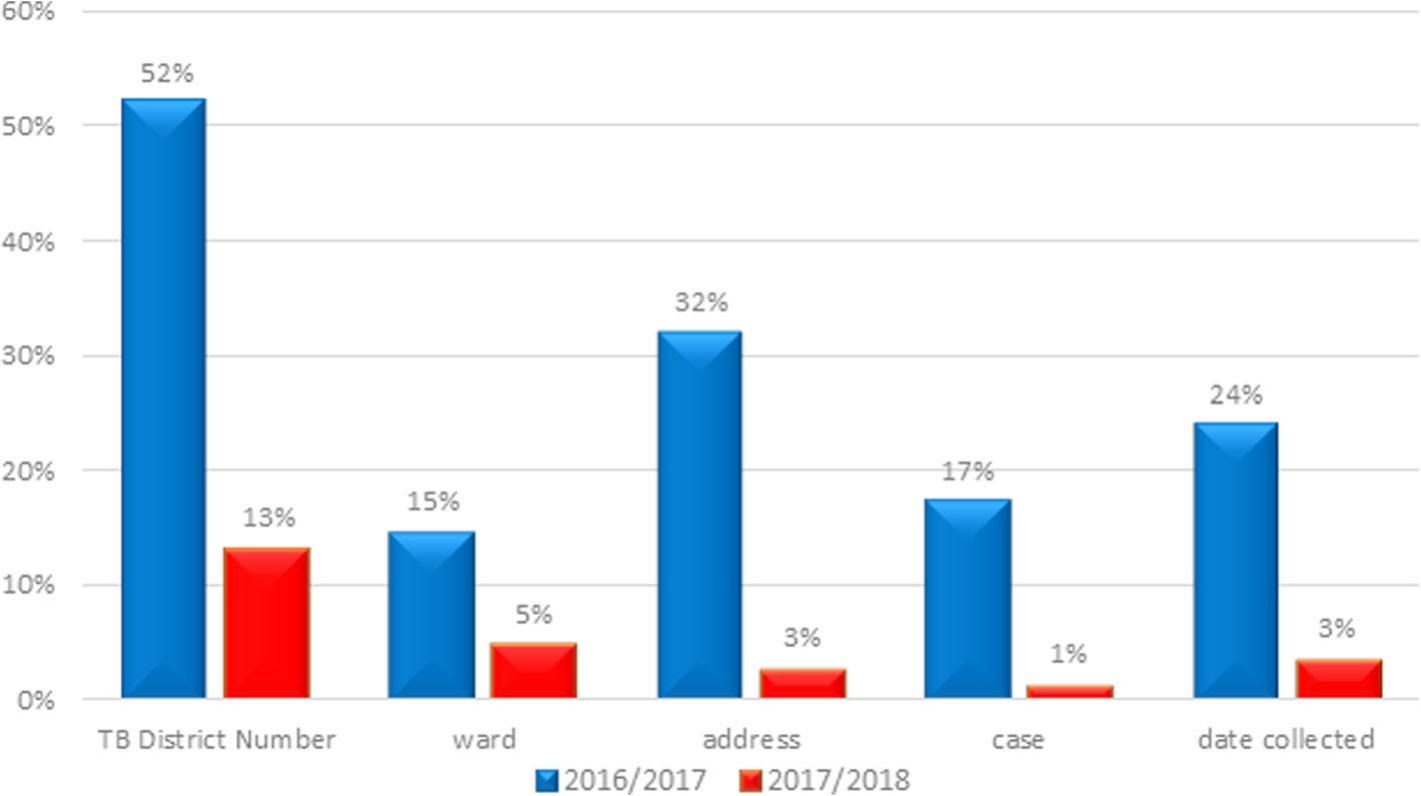
Incomplete TB Laboratory Request Forms 2016/2017 and 2017/2018

Completeness of the filled in TB laboratory request forms received at the CTRL from Mwanza before and after the intervention was compared. In 2016/17 32% had missing address information and 52% had missing district number. In comparison in 2017/18 missing address information dropped to 13% and missing district number fell to 3% (Fig. 5).

**Fig. 6:**
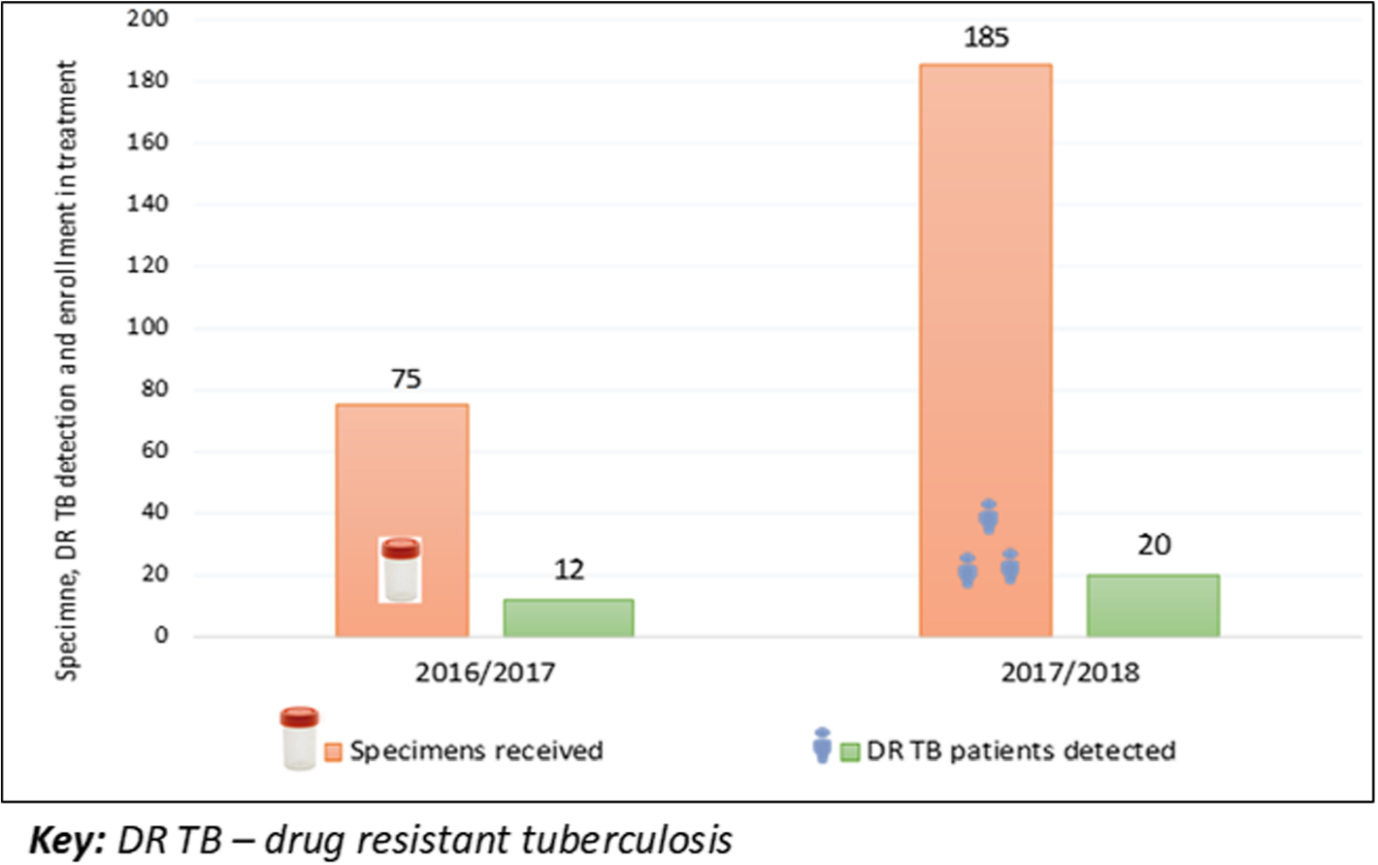
Previously treated drug resistant cases detected (2016/2017 and 2017/2018)

The total annual previously treated cases notified in Tanzania in 2016 were 3072 and in 2017 were 3528. The previously treated cases notified at the National level for Mwanza 2016 was 128 (4%) versus 2017 was 171 (5%), the number of drug resistant TB cases starting treatment increased by 67% (from 12 to 20) (Fig. 6).

## Discussion

The study involved the design and piloting of a revised RSS in Mwanza region. The revised RSS aimed to address many of the issues raised in the earlier qualitative study [12], as well as taking into account the wider availability of molecular testing. The primary outcomes of the study were the impact of the revised RSS on specimen transit time and the time for drug susceptibility results to be sent back to the requester (turnaround time). The pilot of the revised RSS showed significant improvements compared to the current RSS. In particular, reduced transit times and turnaround times for testing which would be expected to lead to drug resistant TB patients starting treatment earlier as well as an increase in the number identified (in this study by around 67%). We observed the volume of previously treated specimens received at the CTRL more than doubled (185 in 2017/18 compared to 75 in 2016/17). The use of local Xpert sites reduced times for specimen transportation in Mwanza region for both sputum and isolates to less than 3 days in most cases.

The transit time recorded for both sputum and isolate specimens in 2017/2018 showed a median of 10 days (IQR 6, 15) with 21% over 21 days. In comparison for sputum specimens in 2016/2017, the median was 12 days (IQR 3, 54) with 44% taking more than 21 days. Unfortunately, due to poor data recording in 2016/2017, the turnaround time is only based on 8 samples where the median turnaround time was 62 days when solid culture was used. By comparison, the use of molecular techniques in 2017/18 saw the turnaround time median drop to 7 days.

In addition, the revised RSS led to 93% of the specimens received at the CTRL being examined in comparison to just 64% in 2016/17 before the revised RSS was implemented. This was supported by a reduction in missing information on laboratory request forms.

Social media played an important role in this study. Various groups created by the NTLP Manager such as Vitendanishi for lab supplies, and the Mwanza family created by the RTLC in Mwanza to enhance timely communication with laboratory personnel, and the DTLC in Mwanza. These groups allowed the ground staff responsible for sending out specimens to resolve concerns timely and eased results dissemination consequently shortening both transit and turnaround times. The platform was replicated in other regions among DTLCs, RTLCs and laboratory teams. Use of WhatsApp groups may also be a useful approach to address other communication issues in the TB programme and beyond.

The study had some limitations due to poor record keeping in the historical data which made some comparisons of performance difficult. However, to a certain degree, this poor record keeping was also symptomatic of the poor performing RSS. The revised RSS record keeping has improved and this is an additional benefit assuming it can be sustained. The evidence from this study shows why this is important as it can have a direct effect on patients getting appropriate treatment and in a timely manner. There was great variability by month in the number of specimens transported for testing (Fig. 4) this was mainly due to operational reasons, for example towards the end of May 2017, there was a nationwide employees’ education certificates inspection for irregularities which led to the termination of several laboratory staff and clinicians. This exercise had a negative impact on the study. In addition, in December 2017 and February 2018, fewer specimens were received which might have been due to staff shortages and some being on holidays leading to some specimen batching.

It is clear from this study there is more to do. For instance, even with the focus of the revised RSS, 13% of laboratory request forms had missing laboratory reference numbers and 3% missing addresses, although much lower than in 2016/17. Similarly, there were significant numbers of specimens in 2017/18 that were not processed for a variety of reasons (i.e. in total 75 out of 472, 15.9%) – see Fig. 3, however, again this is much lower than in 2016/17. Moreover, in the National Strategic Plan (NSP) it was estimated that the MDR TB burdens were 699 and 725 for 2016 and 2017 respectively. The targets set for the region were 349 and 435, but the actual drug resistant TB cases notified were 197 and 200, which implies there remains a large detection gap [17–19].

The study focussed on previously treated cases only, all the interventions looked at could have equal value to new cases, though the level of drug resistance would be expected to be much lower.

## Conclusion

In conclusion, the revised RSS led to an increased number of specimens received and tested at the CTRL. The use of social media within the NTLP network led to close follow-ups and timely response to concerns during the piloting. The revised system was shown to reduce delays in diagnosis and increase in drug resistant case detection. The shorter transit times and turnaround times are important in the diagnosis of MDR-TB and TB control. These positive results suggest a larger scale study involving more regions should be considered to determine whether these benefits are robust and sustainable across similar settings.

## Data Availability

The datasets generated and/or analysed during the current study are available in the figshare repository, 10.6084/m9.figshare.7701458

## Abbreviations

AIDS: Acquired immunodeficiency syndrome
CTRL: Central Tuberculosis Reference Laboratory
DR: Drug Resistant
DRS: Drug Resistant Survey
DR TB: Drug Resistant Tuberculosis
DST: Drug Susceptibility Testing
DTLC: District Tuberculosis and Leprosy Coordinator
EMS: Expedited Mail Services
EPTB: Extra Pulmonary Tuberculosis
FDG: Focus Group Discussion
FLDs: First Line Drugs
HIV: Human immunodeficiency virus
IDI: In Depth Interview
IHI: Ifakara Health Institute
LED: Light Emitting Diode
LJ: Lowenstein Jensen
LPA: Line Probe Assay
LSTM: Liverpool School of Tropical Medicine
MDG: Millennium Development Goals
MDR- TB: Multi-drug resistant tuberculosis
MTB: *Mycobacterium tuberculosis*
MTBC: *Mycobacterium tuberculosis* Complex
NIMR: National Institute for Medical Research
NTM: Non-Tuberculous Mycobacteria
NTLP: National Tuberculosis /Leprosy Programme
NTP: National Tuberculosis Programme
PST: Prevalence Survey Tuberculosis
PTB: Pulmonary Tuberculosis
RA: Researcher Assistant
Retreatment: Previously Treated TB Cases
RLS: Resource Limited Settings
RSS: Routine Surveillance System
RR: Rifampicin Resistance
RR TB: Rifampicin Resistance Tuberculosis
RTLC: Regional Tuberculosis and Leprosy Coordinator
SDGs: Sustainable Development Goals
TB: Tuberculosis
WHO: World Health Organization
XDR: Extensively Drug Resistance
XPERT: GeneXpert MTB RIF
ZN: Ziehl Neelsen

## Footnotes

1 https://select-statistics.co.uk/calculators/sample-size-calculator-two-means/ 15th May 2016
